# Structure, assembly and dynamics of macromolecular complexes by single particle cryo-electron microscopy

**DOI:** 10.1186/1477-3155-11-S1-S4

**Published:** 2013-12-10

**Authors:** Alexandre Durand, Gabor Papai, Patrick Schultz

**Affiliations:** 1 Integrated Structural Biology Department, Institut de Génétique et de Biologie Moléculaire et Cellulaire (IGBMC), U964 Inserm F-67400, UMR7104 CNRS, Université de Strasbourg, 1 Rue Laurent Fries, BP10142, 67404 Illkirch, France

## Abstract

**Background:**

Proteins in their majority act rarely as single entities. Multisubunit macromolecular complexes are the actors in most of the cellular processes. These nanomachines are hold together by weak protein-protein interactions and undergo functionally important conformational changes. TFIID is such a multiprotein complex acting in eukaryotic transcription initiation. This complex is first to be recruited to the promoter of the genes and triggers the formation of the transcription preinitiation complex involving RNA polymerase II which leads to gene transcription. The exact role of TFIID in this process is not yet understood.

**Methods:**

Last generation electron microscopes, improved data collection and new image analysis tools made it possible to obtain structural information of biological molecules at atomic resolution. Cryo-electron microscopy of vitrified samples visualizes proteins in a fully hydrated, close to native state. Molecular images are recorded at liquid nitrogen temperature in low electron dose conditions to reduce radiation damage. Digital image analysis of these noisy images aims at improving the signal-to-noise ratio, at separating distinct molecular views and at reconstructing a three-dimensional model of the biological particle.

**Results:**

Using these methods we showed the early events of an activated transcription initiation process. We explored the interaction of the TFIID coactivator with the yeast Rap1 activator, the transcription factor TFIIA and the promoter DNA. We demonstrated that TFIID serves as an assembly platform for transient protein-protein interactions, which are essential for transcription initiation.

**Conclusions:**

Recent developments in electron microscopy have provided new insights into the structural organization and the dynamic reorganization of large macromolecular complexes. Examples of near-atomic resolutions exist but the molecular flexibility of macromolecular complexes remains the limiting factor in most case. Electron microscopy has the potential to provide both structural and dynamic information of biological assemblies in order to understand the molecular mechanisms of their functions.

## Background

Genomic sequences are now available for many different organisms which, when combined with biocomputing analysis result in the annotation of most of the coding regions that define the protein repertoire of the living creature. Systematic protein purification experiments revealed that proteins act rarely as single entities but are generally associated into well-defined complexes, 80% of which contain between 5 and 12 distinct proteins [1]. Interestingly, several proteins show some degree of infidelity and can be found in distinct complexes. Moreover the documented complexes correspond only to the most stable molecular interactions that resist the harsh protein purification conditions. Many more transient interactions are likely to occur between proteins and protein complexes to build up the intricate and robust molecular interaction network that governs cell fate.

Macromolecular complexes are therefore at the center of most biological processes. They integrate spatially several catalytic or structural activities with built-in regulatory functions. In most of the cases, conformational changes that range from atomic to molecular scale are instrumental to explain the function of these complexes. Altogether these dynamic properties, associated with the size of the particles ranging between 10 and 40 nm substantiates the name of nanomachines often attributed to these complexes. These nanomachines are targeted by most of the currently available drugs used to cure human diseases but for their vast majority the drugs inhibit a catalytic activity carried by a single subunit. Only in rare occasions the intrinsic mechanical properties or the specific protein-protein interaction network of a complex is targeted by drugs. The ribosome is one of such nanomachines, responsible for protein synthesis and for which several examples of drugs targeting the mechanical properties are at hand [2]. Macrolydes and other antibiotics affect the translocation of the ribosome along the mRNA and thus inhibit protein synthesis. Fusidic acid was shown to prevent the dynamic turnover of the elongation factor G and thus affects the interaction of the ribosome with this regulatory factor. Finally antibiotics such as Dalfopristin or Quinopristin were found to bind to the ribosome exit channel and to block mechanically the progression of the nascent polypeptide. Few other examples of drugs targeting so clearly the intrinsic mechanical properties of a complex were described so far. This is related to the poor structural information available to date on complexes since most of the atomic structures deposited in the protein data bank are single polypeptides.

This tutorial aims at describing the molecular organization of the general transcription factor TFIID as a paramount multi-protein complex and to emphasize the role of cryo-electron microscopy (cryo-EM) and digital image analysis to integrate structural and functional information in order to reach a mechanistic model of the complex.

## Methods

### Cryo-EM of frozen hydrated molecular complexes

**Figure 1:**
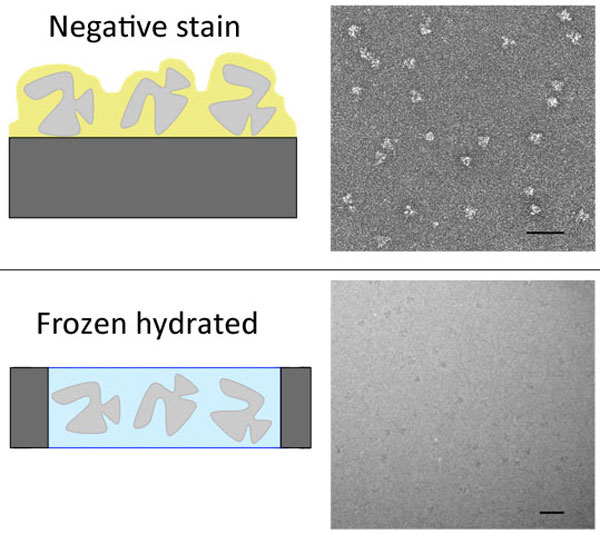
**Preparation of purified molecular complexes for electron microscopy**. In negative stain the specimen is adsorbed on a carbon film, embedded in a layer of heavy metal salts and dried. In frozen hydrated conditions the molecules are embedded in a thin layer of vitrified buffer suspended in a hole of the carbon film. Corresponding electron micrographs are shown (right panels). The bar represents 50 nm.

Imaging of single particles by electron microscopy and numerical analysis of image datasets have proven invaluable tools to describe the structural organization of large macromolecular assemblies. Since the discovery of negative stain by Brenner and Horne in 1959, single particles embedded in a layer of heavy atom salts can be visualized through the high contrast provided by the electron-dense material that surrounds the biological macromolecule composed of low atomic number elements, which poorly scatter electrons (Figure 1) [3]. Despite its ability to provide high contrast, to reveal fine structural details and to sustain fragile structures, the negative staining approach is limited to the description of surface features and rarely extends beyond 15-20 Å resolution.

A major breakthrough was achieved by the discovery in the early 1980' of a robust specimen preparation method that preserved specimen hydration in the vacuum of the electron microscope [4,5]. The method relies on the fast vitrification of a thin aqueous layer containing the specimen by plunging into a liquid ethane slush (Figure 1). This procedure prevents ice crystal formation that segregates particles and ruins image quality. The frozen hydrated sample has to be observed at low temperature, typically close to liquid nitrogen temperature, to prevent phase transitions and special cold stages were developed for cryo-EM observations. This groundbreaking technology opened new horizons for the observation of macromolecular complexes. It allows unconstrained particle conformations in the absence of any crystal contacts and in close to physiological ionic strength and pH conditions. In contrast to crystallized conditions, in which a particular conformation is selected, a flexible particle will be able to adopt all permitted conformations. Conformational flexibility may be detrimental for structure determination since fine structural details may be averaged out, but cryo-EM records conformational intermediates and thus holds the promise to detect and describe particle dynamics. Early electron diffraction experiments showed that in such frozen hydrated conditions, the structure of the specimen is preserved down to atomic resolutions, thus showing for the first time that electron microscopy images have the potential to reveal the same structural information than X-ray diffraction [6].

The resolving power of modern electron microscopes is sufficient to image single atoms. In material sciences, the specimen is very stable and a huge number of electrons can interact with the sample often without affecting its structure. As a result, individual atoms can be detected with a good statistical significance or signal to noise ratio (SNR). Imaging of biological samples fully benefit from the most recent developments in instrumentation such as field emission guns which give a much brighter and more coherent electron beam, detectors and microscope automation. Specific instrumentation is needed to observe frozen hydrated samples which includes cold stages to keep the specimen temperature below -170°C, anti-contamination devices to prevent deposition of traces of water present in the microscope onto the cold specimen as well as low-dose imaging protocols to avoid irradiation of the specimen before data acquisition. Structural damage induced by the electron beam is a strongly limiting factor for biological specimen. Inelastic interactions of incident electrons with sample atoms dissipate energy that can break covalent bonds and generate highly reactive side chains. It is generally accepted that the atomic structure of the specimen is preserved when electron doses are kept below 5 electrons per square angstrom (e^-^/Å^2^), however this number varies with the acceleration voltage of the electrons - at 300 kV it can be up to 25 e^-^/Å^2 ^[7]. In these conditions the molecular images are so noisy that the fine structural details cannot be detected. As a rule of thumb, at an electron dose of 5 e^-^/Å^2^, details in the range of 50 Å can be detected with a SNR of two while smaller details are below this detection limit. To reconcile low specimen irradiation which leads to noisy images, with a high SNR objective to detect small details, it is necessary to split the dose required to detect atoms (say 2000 e^-^/Å^2^) over several independent particles (in this case 400) to kept the dose below 5 e^-^/Å^2 ^and to add-up the signal coming from all these images.

The ongoing development of highly sensitive direct detection cameras and single electron counting devices are important to record highly enlarged images of biological complexes with the best quantum detection efficiency and with reduced noise [8]. Automation of cameras and electron microscopes facilitates the recording of several hundreds to thousands of images per day each containing 100-200 particles thus generating huge image datasets which, as we will describe, will be of importance to reach the final spatial resolution [9]. The need for reduced electron irradiation also led to dedicated "low dose" data acquisition strategies in which microscope adjustments such as focus and astigmatism corrections are performed on an area remote of the area of interest to be recorded "blindly".

### Single particle image analysis

**Figure 2:**
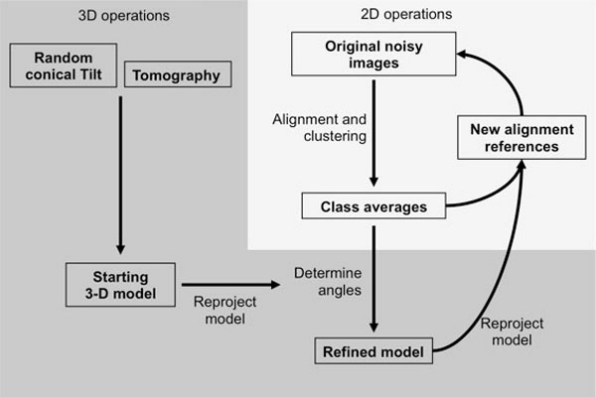
**Schematic representation of the key steps in the analysis of single particle images**.

The objectives of single particle image analysis are dual [10]. The first goal is to improve the SNR of the original images by averaging the signal from independent particles. Assuming a Gaussian distribution of all sources of noise that can affect the original molecular image (statistics of electron-matter interaction, detector noise, cosmic rays, etc...), image averaging will improve the SNR and increase the spatial resolution that can be detected. The second goal of image analysis is to reach a volumetric description of the sample. Standard transmission electron microscopes provide 2-D projections of the 3-D electron density map of the sample, multiplied by a microscope-specific Contrast Transfer Function, which has to be corrected for. The objective is to determine the projection (or viewing) direction of each 2-D image with respect to the 3-D object it originates from and to reconstruct a 3-D model by combining many 2-D views. A brief overview of the image analysis protocol is shown in Figure 2.

#### *Alignment and clustering*

Images of a same particle can be averaged to improve the SNR only if two criteria are met: firstly they have to correspond to the same view or projection of the particle and secondly the images have to be in the same register, or in other words aligned in translation and in rotation one with respect to the others. The spatial resolution that can be reached will depend on the number of images that can be averaged, on how similar the views are, and on the alignment quality. If a tolerance of 10° in viewing direction is accepted, the finest dimension that can be resolved for a globular particle with 10 nm in diameter cannot be smaller than 5.sin10° = 0.8 nm. To reach a resolution of 0.2 nm the variation in viewing direction cannot be larger than 2.2°.

A molecular image is aligned against a reference image by correlating image intensities. The correlation coefficient between two images is a measure of their similarities and all possible translations and rotations will be explored to find the correlation maximum, which will be considered as their best alignment. The quality of the alignment depends on many parameters such as the initial SNR of the image, the size and the shape of the particle.

In a real image data set, the particles have different orientations that lead to different views that need to be separated before calculating an average image. The images need therefore to be clustered into groups containing the most similar images. The image intensity variance should be minimized within the same group, while it should be maximized between different groups. In practical terms the image data set is first subjected to a multivariate statistical analysis (Principal Component Analysis or Correspondence Analysis) to detect the most meaningful trends in the data set and the clustering is then performed on the most significant Eigenvectors using Hierarchical Ascendant Classification schemes.

In an ideal image data set, the particles are randomly oriented which will produce an infinite number of projections. This condition is not always met when particles are adsorbed on a supporting carbon film, which may lead to preferred orientations. Nevertheless, the number of different orientations is very large and it is virtually impossible to find two perfectly identical particle images. It is therefore important to consider an angular projection sector within which we consider the images to be identical at a defined spatial resolution. An image class can to a first approximation be considered as a group of molecular images viewed along the same angular sector. If we consider the above mentioned 10 nm globular particles, a 10° tolerance in projection angle will result in an uncertainty of 0.8 nm. A projection sector of 10° leads to 244 different views and the dataset should be separated in as many classes.

The alignment and clustering steps are highly interdependent and will be used iteratively to improve the quality of the class averages. A better alignment will lead to an improved clustering which will impact the resolution of the class averages. Such high resolution class averages will further improve the alignment of the original images in a multi-reference alignment protocol.

#### *Three-dimensional model building*

The class averages correspond to distinct views of the particle but their projection direction is not known *a priori*. A common-line based method was designed to attribute the relative projection directions of a set of class averages [11], but this method may lead to ambiguous results especially when several conformations of the particle coexist. Two experimental methods, based on the acquisition of tilted images of the same particle have been developed.

In the tomography approach a goniometric electron microscopy stage is used to record tilted views of the same object, typically between +70° and -70° with angular increments of 1 or 2° [12]. After alignment of the images on a common origin, a 3-D model can be calculated for each particle by combining all views for which the exact projection direction is experimentally determined by the position of the tilt axis and the tilt angle. This method suffers from several drawbacks that have been partially addressed. Electron dose and therefore radiolytic damage accumulates during the sequential acquisition of around 140 images of the same particle, but the development of very sensitive low noise cameras restricts the total accumulated dose to 20 to 40 e^-^/Å^2^, enough to reach a resolution of 3-4 nm. The data collection scheme produces a wedge of missing projections and this problem can be overcome by turning the grid 90° in plane and by recording a second tilt series. The missing wedge will then be reduced to a missing pyramid and the quality of the reconstructed volume is generally improved. Alternatively independently reconstructed single particle volumes can be aligned in 3-D and averaged. Since for each orientation of the particle the missing information is different, the averaged volume is essentially devoid of missing wedge artifacts.

In the random conical tilt method, the data collection strategy consists in recording first a 45-60° tilted view of an electron microscopy field containing several particles and, in a second exposure, an untilted view of the same field [13]. The untilted images are analyzed as single particles thus producing classes containing several images of similarly oriented particles each differing by their in-plane or azimuthal angle. This angle, the position of the tilt axis and the tilt angle, informs about the viewing direction of each corresponding tilted image and allows calculating a 3-D model for each class of images. With this data collection strategy, irradiation is limited to a single exposure and the missing information is restricted to a cone.

#### *Model refinement*

The experimental 3-D models are considered as low resolution "starting models" that will be used to determine the viewing direction of independently determined class averages obtained from a much larger image dataset. The starting models will be computationally "reprojected" along many directions to generate a set of reference images of known projection direction. The subsequent alignment of the class averages, or of the original images, against these reprojections in a process called reference-matching will determine the viewing direction for each high resolution class average and lead to an improved, or refined, 3-D model.

#### *Address the dynamic properties of the complexes*

The fast vitrification of the specimen in liquid ethane preserves the hydrated state of the protein complexes, but also cryo-immobilizes their different conformational states. This heterogeneity can hinder high-resolution structure refinement if different conformations are combined in a single class; however it contains essential information about the dynamic properties of the sample. For isolated particles, different conformations can be sorted out computationally when the data set is large enough, thus providing information on mobile parts of the complex. For transient multi-component systems, the relative abundance of the components present in an equilibrium state informs about the interaction constants. It is therefore crucial to detect and separate the conformational states of the specimen both to improve the resolution of each individual state and to describe the dynamics of the examined protein complex. Several methods exist to detect and visualize *de novo *structural heterogeneities in the specimen [14]. Rough movements of domain can be detected by either single-particle tomography or random conical-tilt experiments. More subtle differences can be tracked by using Eigen-analysis of resampled cryo-EM images [15,16]. In this method the images dataset has to be aligned to an average reference structure to determine the relative particle orientation. A large number of volumes is built from a randomly created subset of the dataset and these volumes are subjected to multivariate statistical analysis followed by hierarchal classification to identify the structural differences.

## Results

### The general transcription factor TFIID

Gene expression programs in metazoans are under tight control to achieve growth, development and differentiation of the tissues that make up living organism. A large extend of regulation is performed at the transcriptional level when the information carried by specific DNA sequence (the genes) is transcribed into a messenger RNA molecule (mRNA) by the RNA polymerase II enzyme. Misregulated gene expression underlies many human pathologies, as indicated by germ-line and somatic mutations in transcription regulatory genes that lead to genetic disease [17-20], developmental syndromes[21], neurological diseases [22], epigenetic perturbations [23] and cancer [21,24]. Most intensively studied is the initiation step, which determines which genes are turned on to express a specific piece of genetic information in response to external signaling events. Initiation of transcription is controlled by a large number of multiprotein complexes whose action results in the assembly of a transcription Preinitiation Complex (PIC) on the promoter DNA upstream of the coding sequence. The ultimate goal of the PIC is to position the RNA polymerase II at the transcription start site and to initiate the synthesis of mRNA [25,26].

The general transcription factor TFIID is a key player in the initiation process since it is the first factor to interact with promoter DNA and directs the following steps that result in the onset of transcription [27]. This 1 MDa TFIID multiprotein complex contains a protein recognizing the TATA-box in the gene promoter (TBP) and 13 TBP Associated Factors (TAFs) whose sizes vary between 10 and 250 kDa. To modulate gene expression in response to external signals, small activator or repressor proteins bind upstream of gene promoters and recruit the transcriptional coactivators and the general transcriptional machinery. In this process human TFIID not only recognizes the promoter DNA region of genes but also acts as a transcriptional coactivator by interacting with several such activators like p53, Sp1 and c-Jun [28]. TFIID thus acts as a bridge between transcriptional activator proteins and the PIC.

#### Structure of TFIID, a hybrid approach

How is gene transcription initiated and what is the role of activators and co-activators in this process? How is the activation signal transmitted from the activator to the general transcription factors and finally to the RNA polymerase? How do cells integrate and respond to regulatory signals? What sets different gene expression levels in specific cell types? Which errors in this process lead to disease? To answer these questions the functions of TFIID have to be explained in mechanistic details by determining its biophysical and structural properties.

A large body of structural data is available at atomic scale for single subunit TAF domains and small TFIID sub-assemblies such as the TATA box binding protein [29], the histone-fold containing TAF heterodimers [30-32], the N-terminus of TAF5 [33], The HEAT repeats of TAF6 [34] or the TAF1 bromodomain [35]. Single crystal X-ray diffraction or NMR spectroscopy have determined the atomic structures of parts of TFIID that sum up to about 40% of its total mass but little is known about the organization of these bricks into a functional TFIID assembly that is active in transcription. The full complex is reluctant to crystallization since this large multisubunit complex is difficult to produce in large quantities and with purity suitable for crystal growth. This observation is general to most fields of biology and led to the development of so-called hybrid methods that integrate structural information from different sources. Cryo-electron microscopy is instrumental to this integration since it provides medium (10-20 Å) resolution maps of large complexes into which atomic-scale information obtained by X-ray crystallography or NMR spectroscopy can be fitted [36].

Low-resolution studies by negative-stain [37,38] and, more recently cryo-EM [39] have revealed the general shape of TFIID and allowed approximate localization of several subunits by means of antibody labeling [40,41]. Samples used in these studies were prepared from endogenous sources and resulted in spatial resolutions that were seriously hampered by the dynamic properties, heterogeneous nature and the low abundance of material. The lack of recombinant TFIID complexes of suitable quality and quantity for molecular level studies has been an insurmountable bottleneck to date for structural but also for functional studies. Recent developments in recombinant protein production were instrumental for solving the structure of the core-TFIID complex at 11 Å resolution most probably because several sources of heterogeneity arising from TAF isoforms and posttranslational modifications were reduced [42].

**Figure 3:**
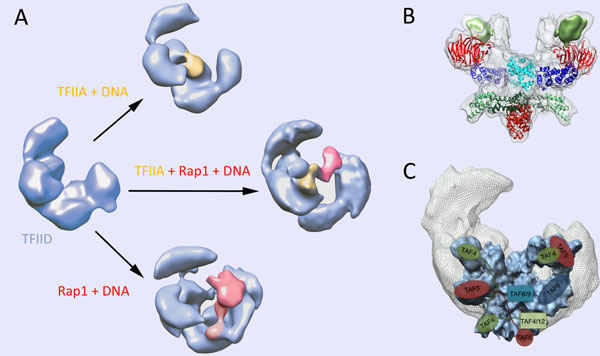
**Structure of TFIID**. (A) Structure of TFIID and of its complexes with TFIIA, DNA and the Rap1 activator. (B) Structure of the core-TFIID at 11.4 Å resolution and fitting of the known atomic structures. (C) Position of the core-TFIID within the complete TFIID complex. The bar represents 7.6 nm in (A), 5 nm in (B) and 5.8 nm in (C)

To gain insights into the function of TFIID, the interaction of yeast TFIID with the promoter DNA was studied in the presence of TFIIA (a general transcription factor required for specific recognition of the TATA element) and the Rap1 activator [43]. The Cryo-EM results revealed the network of interactions and the conformational changes occurring during complex formation. The path of DNA was detected in the complex and these findings extended our understanding on the DNA recognition modalities by TFIID in the presence of trans-acting factors. The resulting structure has shed new light on the intramolecular communication pathways conveying transcription activation signals through the TFIID coactivator (Figure 3). This study revealed an interaction between TFIIA and Rap1 that form a protein bridge between TBP and the DNA-bound Rap1 which results in a large change in the position of TFIIA and of TBP. Interestingly, the concomitant binding of promoter DNA to TFIID-bound Rap1 and to TBP loops out the intervening DNA, thereby accommodating variable distances between Rap1 binding sites and transcription start site.

## Conclusions

The development of cryo-EM and image analysis software has provided new insights into the structural organization and the dynamic reorganization of large macromolecular complexes. Recent improvements in electron microscopy instrumentation allow for automated processing and recording of large image datasets, with an improved image quality due to more stable cold stages and advanced electron optics. With these developments unprecedented close to atomic resolutions were obtained for highly symmetric biological assemblies such as icosahedral viruses. It can be anticipated that the analysis of large datasets as well as new data acquisition strategies that compensate for particle movement during acquisition, will routinely provide molecular models better than 5A in the near future. The analysis of molecular flexibility still requires algorithmic developments to describe concomitantly the high resolution structure and the continuous conformational space of a macromolecular complex. The unique asset of Cryo-EM however resides in the possibility to record images of single particles which collectively contain both structural and dynamic information.

## Competing interests

The authors declare that they have no competing interests.

## Authors' contributions

P.S. initiated the study. GP performed the electron microscopy and image analysis experiments. The manuscript was prepared and commented on by P.S., A.D. and G.P.

## References

[B1] Gavin AC Bosche M Krause R Grandi P Marzioch M Bauer A Schultz J Rick JM Michon AM Cruciat CM Functional organization of the yeast proteome by systematic analysis of protein complexes Nature 2002 11 6868 141 147 10.1038/415141a 11805826

[B2] Wilson DN On the specificity of antibiotics targeting the large ribosomal subunit Ann N Y Acad Sci 2011 11 1 16 10.1111/j.1749-6632.2011.06192.x 22191523

[B3] Brenner S Horne RW A negative staining method for high resolution electron microscopy of viruses Biochim Biophys Acta 1959 11 103 110 13804200 10.1016/0006-3002(59)90237-9

[B4] Lepault J Booy FP Dubochet J Electron microscopy of frozen biological suspensions J Microsc 1983 11 Pt 1 89 102 6186816 10.1111/j.1365-2818.1983.tb04163.x

[B5] Dubochet J Adrian M Chang JJ Homo JC Lepault J McDowall AW Schultz P Cryo-electron microscopy of vitrified specimens Q Rev Biophys 1988 11 2 129 228 10.1017/S0033583500004297 3043536

[B6] Taylor KA Glaeser RM Electron diffraction of frozen, hydrated protein crystals Science 1974 11 4168 1036 1037 10.1126/science.186.4168.1036 4469695

[B7] Glaeser RM Retrospective: radiation damage and its associated "information limitations" J Struct Biol 2008 11 3 271 276 10.1016/j.jsb.2008.06.001 18588985

[B8] Henderson R McMullan G Problems in obtaining perfect images by single-particle electron cryomicroscopy of biological structures in amorphous ice J Electron Microsc (Tokyo) 2013 11 1 43 50 10.1093/jmicro/dfs094 PMC3767125 23291269

[B9] Carragher B Kisseberth N Kriegman D Milligan RA Potter CS Pulokas J Reilein A Leginon: an automated system for acquisition of images from vitreous ice specimens J Struct Biol 2000 11 1 33 45 10.1006/jsbi.2000.4314 11121305

[B10] Orlova EV Saibil HR Structure determination of macromolecular assemblies by single-particle analysis of cryo-electron micrographs Curr Opin Struct Biol 2004 11 5 584 590 10.1016/j.sbi.2004.08.004 15465319

[B11] Van Heel M Angular reconstitution: a posteriori assignment of projection directions for 3D reconstruction Ultramicroscopy 1987 11 2 111 123 10.1016/0304-3991(87)90078-7 12425301

[B12] Walz J Typke D Nitsch M Koster AJ Hegerl R Baumeister W Electron Tomography of Single Ice-Embedded Macromolecules: Three-Dimensional Alignment and Classification J Struct Biol 1997 11 3 387 395 10.1006/jsbi.1997.3934 9441941

[B13] Radermacher M Three-dimensional reconstruction of single particles from random and nonrandom tilt series J Electron Microsc Tech 1988 11 4 359 394 10.1002/jemt.1060090405 3058896

[B14] Leschziner AE Nogales E Visualizing flexibility at molecular resolution: analysis of heterogeneity in single-particle electron microscopy reconstructions Annu Rev Biophys Biomol Struct 2007 11 43 62 10.1146/annurev.biophys.36.040306.132742 17201674

[B15] Simonetti A Marzi S Myasnikov AG Fabbretti A Yusupov M Gualerzi CO Klaholz BP Structure of the 30S translation initiation complex Nature 2008 11 7211 416 420 10.1038/nature07192 18758445

[B16] Penczek PA Kimmel M Spahn CM Identifying conformational states of macromolecules by eigen-analysis of resampled cryo-EM images Structure 2011 11 11 1582 1590 10.1016/j.str.2011.10.003 22078558 PMC3255080

[B17] Compe E Egly JM TFIIH: when transcription met DNA repair Nat Rev Mol Cell Biol 2012 11 6 343 354 10.1038/nrm3350 22572993

[B18] Fuss JO Tainer JA XPB and XPD helicases in TFIIH orchestrate DNA duplex opening and damage verification to coordinate repair with transcription and cell cycle via CAK kinase DNA Repair (Amst) 2011 11 7 697 713 10.1016/j.dnarep.2011.04.028 21571596 PMC3234290

[B19] Helmlinger D Tora L Devys D Transcriptional alterations and chromatin remodeling in polyglutamine diseases Trends Genet 2006 11 10 562 570 10.1016/j.tig.2006.07.010 16911843

[B20] Matsumoto T Sakari M Okada M Yokoyama A Takahashi S Kouzmenko A Kato S The androgen receptor in health and disease Annu Rev Physiol 2013 11 201 224 10.1146/annurev-physiol-030212-183656 23157556

[B21] Fadloun A Kobi D Pointud JC Indra AK Teletin M Bole-Feysot C Testoni B Mantovani R Metzger D Mengus G The TFIID subunit TAF4 regulates keratinocyte proliferation and has cell-autonomous and non-cell-autonomous tumour suppressor activity in mouse epidermis Development 2007 11 16 2947 2958 10.1242/dev.005041 17626060

[B22] Hashimoto S Boissel S Zarhrate M Rio M Munnich A Egly JM Colleaux L MED23 mutation links intellectual disability to dysregulation of immediate early gene expression Science 2011 11 6046 1161 1163 10.1126/science.1206638 21868677

[B23] Duncan EM Allis CD Errors in erasure: links between histone lysine methylation removal and disease Prog Drug Res 2011 11 69 90 21141725 10.1007/978-3-7643-8989-5_4

[B24] Kalogeropoulou M Voulgari A Kostourou V Sandaltzopoulos R Dikstein R Davidson I Tora L Pintzas A TAF4b and Jun/activating protein-1 collaborate to regulate the expression of integrin alpha6 and cancer cell migration properties Mol Cancer Res 2010 11 4 554 568 10.1158/1541-7786.MCR-09-0159 20353996

[B25] Orphanides G Lagrange T Reinberg D The general transcription factors of RNA polymerase II Genes Dev 1996 11 21 2657 2683 10.1101/gad.10.21.2657 8946909

[B26] Roeder RG The role of general initiation factors in transcription by RNA polymerase II Trends Biochem Sci 1996 11 9 327 335 8870495

[B27] Papai G Weil PA Schultz P New insights into the function of transcription factor TFIID from recent structural studies Curr Opin Genet Dev 2011 11 2 219 224 10.1016/j.gde.2011.01.009 21420851 PMC3081712

[B28] Liu WL Coleman RA Ma E Grob P Yang JL Zhang Y Dailey G Nogales E Tjian R Structures of three distinct activator-TFIID complexes Genes Dev 2009 11 13 1510 1521 10.1101/gad.1790709 19571180 PMC2704470

[B29] Nikolov DB Hu SH Lin J Gasch A Hoffmann A Horikoshi M Chua NH Roeder RG Burley SK Crystal structure of TFIID TATA-box binding protein Nature 1992 11 6399 40 46 10.1038/360040a0 1436073

[B30] Birck C Poch O Romier C Ruff M Mengus G Lavigne AC Davidson I Moras D Human TAF(II)28 and TAF(II)18 interact through a histone fold encoded by atypical evolutionary conserved motifs also found in the SPT3 family Cell 1998 11 2 239 249 10.1016/S0092-8674(00)81423-3 9695952

[B31] Werten S Mitschler A Romier C Gangloff YG Thuault S Davidson I Moras D Crystal structure of a subcomplex of human transcription factor TFIID formed by TATA binding protein-associated factors hTAF4 (hTAF(II)135) and hTAF12 (hTAF(II)20) J Biol Chem 2002 11 47 45502 45509 10.1074/jbc.M206587200 12237304

[B32] Gangloff YG Pointud JC Thuault S Carre L Romier C Muratoglu S Brand M Tora L Couderc JL Davidson I The TFIID components human TAF(II)140 and Drosophila BIP2 (TAF(II)155) are novel metazoan homologues of yeast TAF(II)47 containing a histone fold and a PHD finger Mol Cell Biol 2001 11 15 5109 5121 10.1128/MCB.21.15.5109-5121.2001 11438666 PMC87236

[B33] Romier C James N Birck C Cavarelli J Vivares C Collart MA Moras D Crystal structure, biochemical and genetic characterization of yeast and E. cuniculi TAF(II)5 N-terminal domain: implications for TFIID assembly J Mol Biol 2007 11 5 1292 1306 10.1016/j.jmb.2007.02.039 17397863

[B34] Scheer E Delbac F Tora L Moras D Romier C TFIID TAF6-TAF9 complex formation involves the HEAT repeat-containing C-terminal domain of TAF6 and is modulated by TAF5 protein J Biol Chem 2012 11 33 27580 27592 10.1074/jbc.M112.379206 22696218 PMC3431708

[B35] Jacobson RH Ladurner AG King DS Tjian R Structure and function of a human TAFII250 double bromodomain module Science 2000 11 5470 1422 1425 10.1126/science.288.5470.1422 10827952

[B36] Lander GC Saibil HR Nogales E EM, crystallography, and beyond Curr Opin Struct Biol 2012 11 5 627 635 Go hybrid 10.1016/j.sbi.2012.07.006 22835744 PMC3478466

[B37] Brand M Leurent C Mallouh V Tora L Schultz P Three-dimensional structures of the TAFII-containing complexes TFIID and TFTC Science 1999 11 5447 2151 2153 10.1126/science.286.5447.2151 10591645

[B38] Andel F Ladurner AG Inouye C Tjian R Nogales E Three-dimensional structure of the human TFIID-IIA-IIB complex Science 1999 11 5447 2153 2156 10.1126/science.286.5447.2153 10591646

[B39] Papai G Tripathi MK Ruhlmann C Werten S Crucifix C Weil PA Schultz P Mapping the initiator binding Taf2 subunit in the structure of hydrated yeast TFIID Structure 2009 11 3 363 373 10.1016/j.str.2009.01.006 19278651 PMC2677412

[B40] Leurent C Sanders SL Demeny MA Garbett KA Ruhlmann C Weil PA Tora L Schultz P Mapping key functional sites within yeast TFIID EMBO J 2004 11 4 719 727 10.1038/sj.emboj.7600111 14765106 PMC381015

[B41] Leurent C Sanders S Ruhlmann C Mallouh V Weil PA Kirschner DB Tora L Schultz P Mapping histone fold TAFs within yeast TFIID EMBO J 2002 11 13 3424 3433 10.1093/emboj/cdf342 12093743 PMC126091

[B42] Bieniossek C Papai G Schaffitzel C Garzoni F Chaillet M Scheer E Papadopoulos P Tora L Schultz P Berger I The architecture of human general transcription factor TFIID core complex Nature 2013 11 7434 699 702 10.1038/nature11791 23292512

[B43] Papai G Tripathi MK Ruhlmann C Layer JH Weil PA Schultz P TFIIA and the transactivator Rap1 cooperate to commit TFIID for transcription initiation Nature 2010 11 7300 956 960 10.1038/nature09080 20559389 PMC2900199

